# Clinical Utility of a Multiplex PCR Panel (BioFire Joint Infection^®^) in the Adjustment of Empiric Antimicrobial Therapy: Experience in Pediatric Osteoarticular Infections

**DOI:** 10.3390/children11101236

**Published:** 2024-10-14

**Authors:** Clara Udaondo, Rosa María Alcobendas Rueda, Blanca Diaz-Delgado, Agustin Remesal, Inmaculada Quiles-Melero, Cristina Calvo

**Affiliations:** 1 Pediatric Rheumatology, Hospital Universitario La Paz, 28046 Madrid, Spain; 2 CIBERINFEC, Consorcio de Investigación Biomédica en Red, Hospital Carlos III, 28029 Madrid, Spain; 3 Pediatric Rheumatology Unit, University Hospital Ruber International, 28034 Madrid, Spain; 4 Clinical Microbiology and Parasitology Department, Hospital Universitario La Paz, 28046 Madrid, Spain; 5 Pediatric Infectious Diseases Department, Hospital Universitario La Paz, 28046 Madrid, Spain

**Keywords:** pediatric, osteoarticular infections, FilmArray, diagnosis, PCR

## Abstract

Background/Objectives: This study aims to evaluate the impact of the PCR multiplex panel (BioFire JI^®^) on the diagnosis and management of pediatric osteoarticular infections. Methods: This retrospective study analyzed data from pediatric patients diagnosed with osteoarticular infections between January 2023 and April 2024. The effectiveness of the PCR multiplex panel in identifying pathogens was compared with traditional culture methods. Results: In total, 50 patients were identified (66.6% male, 74% under 3 years of age). They were diagnosed as follows: septic arthritis in 46%, osteomyelitis in 26%, and septic osteoarthritis in 22%. An identifiable agent was isolated by conventional culture in 22 cases (44%). *Kingella kingae* was the predominant pathogen identified, accounting for 50% of cases (11/22), followed by *Staphylococcus aureus* (9/22). The BioFire JI^®^ Panel PCR demonstrated a sensitivity of 93%, with a specificity of 63% when evaluated against synovial fluid culture as the reference standard. The panel identified seven additional pathogens not detected by conventional culture methods: 2/9 MSSA (22%), 1/1 *S. pyogenes* (100%), and 4/11 *K. kingae* (37%), increasing the yield by 14%. The rapid identification of pathogens facilitated timely and targeted therapeutic interventions. Conclusions: The PCR multiplex panel (BioFire JI^®^) improved the diagnosis of pediatric osteoarticular infections.

## 1. Introduction

Pediatric osteoarticular infections (OAIs) are serious medical conditions that, if not promptly diagnosed and treated, may result in severe morbidity and long-term disabilities [1]. The most commonly implicated bacteria in pediatric OAIs are *Staphylococcus aureus*, followed by *Kingella kingae* [2,3,4]. *S. aureus* is associated with more complications and a worse outcome than *K. kingae*, which exhibits a milder clinical course and typically appears in young children [5,6,7].

Exclusively oral therapy in selected cases of *K. kingae* infections minimizes unnecessary hospital admissions and the need for intravenous antibiotic therapy, alleviating the discomfort experienced by pediatric patients and their families [5,8]. By identifying the specific pathogen driving the infection, treatment can be precisely targeted, optimizing efficacy while minimizing the risk of antimicrobial resistance.

In recent years, molecular detection techniques have made significant progress in identifying the causative agents of OAI, improving diagnostic yield and allowing rapid and accurate identification of pathogens at the earliest stages of decision making [6,7]. The BioFire^®^ Joint Infection (JI) Panel is a multiplex polymerase chain reaction (PCR)-based system designed to identify 29 types of Gram-positive and Gram-negative bacteria, along with two yeast species and eight common antibiotic resistance genes associated with osteoarticular infections. Results are available within one hour, enabling timely clinical decision making and optimizing patient care [9,10].

The objective of this study is to describe the impact of the BioFire^®^JI panel on diagnostic accuracy and subsequent clinical management of pediatric osteoarticular infections in our hospital.

## 2. Materials and Methods

### 2.1. Design

This retrospective, single-center study involved a comprehensive review of medical records.

### 2.2. Sample

Inclusion criteria encompassed patients under the age of 18 who were seen at Hospital La Paz (Madrid, Spain) between January 2023 and April 2024 with a diagnosis of septic arthritis (SA), septic osteoarthritis (SOA), osteomyelitis (OM), spondylodiscitis (SD), or pyogenic sacroiliitis (PSI).

### 2.3. Definitions

Osteomyelitis (OM) was defined as (1) the presence of clinical features (fever, pain, restriction of movement), (2) confirmed by radiological study that identified the location of the infection (bone scan, magnetic resonance imaging (MRI), computerized tomography (CT) or ultrasound), with or without (3) bacterial isolation from blood or bone sample. Spondylodiscitis (SD) and pyogenic sacroiliitis (PSI) were included in this definition. 

Confirmed septic arthritis (SA) was defined as (1) the presence of clinical features (fever, joint swelling, functional disability or pain), with (2) joint effusion demonstrated by ultrasound or by physical examination performed by an experienced physician, and (3) bacterial isolation from joint fluid or blood culture [11]. Probable SA was considered when, in the absence of microbiological isolation, there was a favorable response to antibiotic treatment with a minimum of 6 months follow-up, and if joint fluid was obtained, it showed more than 40,000 cells/mm^3^. A lower count than the classical 50,000 cells/mm^3^ was chosen to improve the sensitivity of SA recruitment. 

Septic osteoarthritis (SOA) was considered when the infection met criteria for both entities simultaneously.

### 2.4. Management of Pediatric Osteoarticular Infections

According to our hospital protocols, blood culture, synovial fluid, and abscess pus were taken and sent for culture if possible. Additionally, if there was enough sample, the BioFire^®^JI Panel was performed on synovial fluid and/or abscess pus when applicable. The BioFire^®^ JI Panel (BioMérieux SA, BioFire Diagnostics, LLC, Salt Lake City, UT, USA) was approved by the FDA and CE marked. BioFire^®^JI analysis was performed according to the manufacturer’s guidelines.

Following the isolation of a causative pathogen, clinical decisions were made according to our guidelines, offering exclusively oral treatment from the onset for patients who met the low risk criteria (good general condition, no underlying disease, 6 months to 3 years old, appropriate oral tolerance, C-reactive protein <80 mg/L, erythrocyte sedimentation rate/C-reactive protein ratio ≥0.67, no skin injury, no recent surgery, no cervical spondylodiscitis, and no local complications at onset) [12]. The remaining patients were admitted and started on intravenous treatment with a subsequent switch to oral therapy once clinical and analytical improvement was confirmed [11,12].

### 2.5. Statistical Analysis

A description of the data was performed. Quantitative data were expressed as mean and standard deviation or median and interquartile range, where appropriate. Qualitative data were expressed as absolute frequencies and percentages. Sensitivity, specificity, and the positive predictive value of PCR were calculated in relation to synovial culture.

## 3. Results

A total of 50 pediatric patients with osteoarticular infections were included in this study, with a slight male predominance (66.6%). Thirty-seven children (74%) were aged under 3 years (median age: 14 months, IQR 11–18.50), and thirteen (26%) were aged over 3 years (median age: 10.5 years, IQR 6–12). SA was the most common diagnosis (46%; 23/50—15 confirmed, 8 probable), followed by OM (26%; 13/50) and SOA (11/50, 22%; 6 confirmed, 5 probable). There were two cases of SD and one case of PSI, all in children below 3 years of age. Table 1. 

### 3.1. Microbiology

Of the 50 osteoarticular infections, an identifiable agent was isolated by conventional culture in 22 cases, while 28 had no microbiological confirmation (12 OM, 8 SA, 5 SAO, 2 SD, and 1 PSI). *K. kingae* was the predominant pathogen identified, accounting for 50% of cases (11/22), followed by *S. aureus* (9/22, 41%), all methicillin-sensitive (MSSA), *Streptococcus pyogenes*, and *Haemophilus influenzae* (1/22, 4.5% each). Notably, all *K. kingae* isolates were from children under 3 years of age, while *S. pyogenes* and 8/9 *S. aureus* were found in children older than 3 years.

The results of the samples sent to microbiology are detailed in Table 1. No local sample could be obtained for 20 patients (12 OM, 4 SA, 1 SOA, 2 SD, and 1 PSI). The BioFire^®^ JI panel was performed in 30 cases: 11 were negative, 8 identified *S. aureus*, 1 identified *S. pyogenes*, and 10 identified *K. kingae*. Of these, four had received previous antibiotic treatment. Among the 11 cases in which the panel was negative (6 SA, 5 SOA), both laboratory tests and clinical evolution were compatible with OAI.

The BioFire^®^ JI panel identified seven pathogens not detected by conventional culture methods: 2/9 MSSA (22%), 1/1 *S. pyogenes* (100%), and 4/11 *K. kingae* (37%), increasing the yield by 14%.

The BioFire JI Panel PCR demonstrated a sensitivity of 93%, specificity of 63%, positive predictive value of 68%, and negative predictive value of 91% when evaluated against synovial fluid culture as the reference standard.

### 3.2. Arthrocentesis

Among the 34 patients with arthritis (23 SA and 11 SOA), there were 3 cases involving shoulders, 3 elbows, 11 hips, 13 knees, 3 ankles, and 1 metatarsophalangeal joint. Arthrocentesis was performed in 27 cases, while in 7 cases it was not performed: 1 shoulder, 1 elbow, and 5 hips.

Among the seven cases where arthrocentesis was not performed, two underwent surgery, while in five cases no intervention was carried out due to minimal joint effusion (one shoulder, three hips initially diagnosed with OM with minimal associated effusion, and one elbow). The average number of arthrocentesis procedures was 1.59. Of the 27 patients who underwent arthrocentesis, all samples were sent for microbiologic analysis (culture +/− panel). In only 18 patients was there enough sample to send for cytochemical analysis, with a median cell count of 74,255 (IQR: 27,892–133,410).

### 3.3. Treatment

Treatment modalities varied depending on the clinical presentation and pathogen identified. Empirical antibiotic therapy was initiated in all cases, based on the international guidelines and our PROA recommendations [12,13]. In those with initial intravenous therapy, cefuroxime + clindamycin was started in 13 cases and cloxacillin + clindamycin in 8 cases. 

Twenty-seven patients (54%) started on oral treatment at baseline, twenty-five of them meeting low-risk criteria (7/11 *K. kingae*, 4/11sterile) and two of them because they had already started oral treatment before the first visit to our center and had shown good clinical evolution (2 *S. aureus*). All of them had good clinical outcomes. Oral antibiotic treatment was cefuroxime axetil in 12/27 cases, amoxicillin clavulanate in 8/27 cases and cefadroxil in 7/27 cases. In the two cases of *S. Aureus*, oral clindamycin was added initially. 

One patient had bilateral arthritis due to *K. kingae*; despite ruling out endocarditis and systemic complications (normal chest X-ray, scintigraphy, and fundus examination), this patient received intravenous treatment for 28 days. Transition to oral antibiotics occurred in all cases, primarily guided by clinical improvement and laboratory parameters. The median duration of intravenous therapy was 7 days (range 2–21), and the total duration of antibiotic treatment was 38 days (range 15–84 days).

Surgical intervention was necessary in eight cases (16%), predominantly for drainage of purulent material and debridement of infected tissues (2 SA, 5 SOA, 1 OM). All except one were in children older than 3 years. The pathogens involved were *S. aureus* in five cases, *S. pyogenes* in one, and *H. influenzae* in one.

## 4. Discussion

The use of PCR in detecting microorganisms in osteoarticular infections has several advantages over traditional culture methods [14]. PCR can detect microorganisms in culture-negative samples, which is especially useful in cases where patients have been previously treated with antibiotics, as was shown in a study by Levy et al. where systematic detection of 16S rDNA in negative-culture samples led to an additional 9% diagnosis [15]. The assay was unaffected by prior antibiotic use, which was the case in 4 patients in our series, overcoming a significant limitation of conventional culture methods [16]. Furthermore, it was able to detect difficult-to-culture organisms: in our series, 5/11 cases of *K. kingae* were detected only by PCR, with negative blood and synovial cultures.

The enhanced diagnostic yield offered by these modalities empowers clinicians to expedite targeted therapy initiation, reducing the need for prolonged hospitalizations. In a recent clinical trial, in 248 children and adolescents with uncomplicated bone and joint infections, initial oral antibiotic treatment was non-inferior to initial intravenous antibiotics followed by oral therapy [17]. Moreover, early confirmation of *K. kingae* often leads to outpatient management with oral antibiotics, improving patient comfort and resource utilization, given the growing evidence of the good evolution with oral antibiotic therapy in this infection [4,5,6]. Conversely, the identification of *S.aureus* allows immediate targeted therapy—with meticillin resistance genes included in the BioFire^®^JI panel—even before culture results confirm antibiotic sensitivity, ensuring close monitoring due to the higher risk of complications [18].

Integrating such cutting-edge technologies into routine practice streamlines diagnostic workflows and facilitates timely interventions, particularly for suspected *K. kingae* OAI fulfilling low-risk criteria. This proactive approach promotes outpatient management when feasible, optimizing resources and improving patient satisfaction [8,17,19]. However, PCR should complement and not replace culture methods, to ensure specificity and accuracy in diagnosing osteoarticular infections [15].

Our study has some limitations. Joint fluid samples could not be obtained in all cases, complicating the identification of the causative agent in patients with OM, SD, or PSI who did not require surgery. Furthermore, the panel returned negative results in 10 cases where laboratory tests and clinical presentation were consistent with OAI. These cases were treated based on clinical suspicion despite the lack of microbiological confirmation. Additionally, the sample size of our study was small and larger studies are needed to confirm our results.

Our review underscores the importance of obtaining synovial fluid samples for diagnosis and the evolving landscape of surgical interventions in pediatric OAI. While surgical debridement remains essential for poor prognosis or complications, a judicious approach should be reserved for cases where conservative management is inadequate.

## 5. Conclusions

The multiplex PCR BIOFIRE^®^JI panel is a promising complementary tool for identifying causative agents in OAI, alongside traditional culture methods. It increases diagnostic yield by detecting pathogens not identified by conventional cultures and offers rapid results, even in patients who have received prior antibiotic therapy. Its implementation in daily practice aids in quick decision making regarding antibiotic therapy regimens and anticipating potential complications, especially for pathogens associated with poorer prognoses.

## Figures and Tables

**Table 1:** Microbiological results.

Agent	N	Diagnosis	Blood Culture	Synovial Fluid Culture	BIOFIRE FilmArray	Only Detected by FilmArray
*K. kingae*	11 patients	11 SA	1/10	6/10	10/10	4
*S. aureus*	9 patients	1 OM 4 SA 4 SOA	2/8	7/8	8/9	2
*S. pyogenes*	1 patient	1 SOA	0/1	0/1	1/1	1
*H. influenzae*	1 patient	1 SOA	0/1	1/1	0/1	0
Unknown	28 patients	12 OM 8 SA 5 SOA 2 SD 1 PSI	0/21	0/9	0/9	

SA: septic arthritis, OM: osteomyelitis, SOA: septic ostheoarthritis, SD: spondylodiscitis, PSI: pyogenic sacroiliitis.

## Data Availability

The data are not publicly available due to technical and time reasons.
